# Presepsin and prognostic nutritional index are predictors of septic acute kidney injury, renal replacement therapy initiation in sepsis patients, and prognosis in septic acute kidney injury patients: a pilot study

**DOI:** 10.1186/s12882-021-02422-x

**Published:** 2021-06-12

**Authors:** Yuichiro Shimoyama, Osamu Umegaki, Noriko Kadono, Toshiaki Minami

**Affiliations:** 1 Department of Anesthesiology, Osaka Medical College, Intensive Care Unit, Osaka Medical College Hospital, 2-7 Daigaku-machi, Takatsuki, Osaka, 569-8686 Japan; 2 Department of Anesthesiology, Osaka Medical College, Osaka Medical College Hospital, Takatsuki, Japan

**Keywords:** Presepsin, Sepsis, Sepsis-3 definition, Inflammation-based prognostic scores, Acute kidney injury, Renal replacement therapy

## Abstract

**Background:**

Sepsis is the most common cause of acute kidney injury (AKI) among critically ill patients. This study aimed to determine whether presepsin is a predictor of septic acute kidney injury, renal replacement therapy initiation (RRTi) in sepsis patients, and prognosis in septic AKI patients.

**Methods:**

Presepsin values were measured immediately after ICU admission (baseline) and on Days 2, 3, and 5 after ICU admission. Glasgow Prognostic Score (GPS), neutrophil to lymphocyte ratio (NLR), platelet to lymphocyte ratio, Prognostic Index, and Prognostic Nutritional Index (PNI) were measured at baseline, and total scores (“inflammation-presepsin scores [iPS]”) were calculated for category classification. Presepsin values, inflammation-based prognostic scores, and iPS were compared between patients with and without septic AKI or RRTi and between survivors and non-survivors.

**Results:**

Receiver operating characteristic curve analyses identified the following variables as predictors of septic AKI and RRTi in sepsis patients: presepsin on Day 1 (AUC: 0.73) and Day 2 (AUC: 0.71) for septic AKI, and presepsin on Day 1 (AUC: 0.71), Day 2 (AUC: 0.9), and Day 5 (AUC: 0.96), Δpresepsin (Day 2 – Day 1) (AUC: 0.84), Δpresepsin (Day 5 – Day 1) (AUC: 0.93), and PNI (AUC: 0.72) for RRTi. Multivariate logistic regression analyses identified presepsin on Day 2 as a predictor of prognosis in septic AKI patients.

**Conclusions:**

Presepsin and PNI were found to be predictors of septic AKI, RRTi in sepsis patients, and prognosis in septic AKI patients.

## Background

Sepsis is the main cause of mortality in critically ill intensive care unit (ICU) patients [1], and new sepsis criteria were established in 2016 [2]. Sepsis is the most common cause of acute kidney injury (AKI) among critically ill patients [3]. Early treatment with appropriate antibiotics improves the prognosis and survival of severe sepsis and septic shock patients [4–6].

Procalcitonin (PCT) is the main biomarker used to diagnose sepsis [7], but its values increase in non-sepsis pathologies as well [8–10]. Presepsin is a subtype of soluble CD14 (CD14-ST) [11], and is an accurate biomarker for diagnosing sepsis. Presepsin has a higher specificity for sepsis diagnosis compared with PCT and IL-6 [12], and thus could be useful for the prognosis of sepsis and monitoring the course of the disease [13]. Another advantage of presepsin is that it can be measured in less than 17 min with a compact fully automated immunoanalyzer (PATHFAST®; Mitsubishi Chemical Medience Corporation, Tokyo, Japan) [14].

The Glasgow Prognostic Score (GPS; based on serum C-reactive protein (CRP) and albumin levels), neutrophil to lymphocyte ratio (NLR), platelet to lymphocyte ratio (PLR), Prognostic Nutritional Index (PNI; based on albumin and lymphocyte counts), and the Prognostic Index (PI; based on serum CRP and white blood cell counts), are inflammation-based prognostic scores which are useful prognostic biomarkers for many types of cancer [15]. However, no study has investigated the association of septic acute kidney injury (AKI), renal replacement therapy initiation (RRTi), and prognosis of septic AKI with presepsin values alone or in combination with the above-mentioned inflammation-based prognostic scores in septic ICU patients.

The present study aimed to prove the following hypotheses: 1) presepsin can predict septic AKI, RRTi in sepsis ICU patients, and prognosis in septic AKI ICU patients; and 2) the ability of presepsin to predict the above are superior to inflammation-based prognostic scores and can be improved by combining presepsin values with inflammation-based prognostic scores.

## Methods

### Patients and study design

The study design, inclusion and exclusion criteria, and definition of “inflammation-presepsin scores [iPS]” used in the present study were published previously [16]. Septic AKI was defined as stage ≥1 kidney disease according to the Kidney Disease: Improving Global Outcomes (KDIGO) classification [17]. The need for RRTi was determined according to the KDIGO classification (i.e., stage > 3 kidney disease). Presepsin values, inflammation-based prognostic scores, iPS, and changes (Δ) in presepsin values relative to baseline values at each sampling point were compared between patients with and without septic AKI or RRTi and between survivors and non-survivors.

### Laboratory assessments

Presepsin concentration was measured by PATHFAST® (Mitsubishi Chemical Medience Corporation, Tokyo, Japan) [14]. Threshold values were as follows: (a) 300 to 500 pg/ml: “systemic infection (sepsis) possible”; (b) 500 to 1000 pg/ml: “significant risk of systemic infection progression (severe sepsis), increased risk of unfavorable outcome”; and (c) ≥1000 pg/ml: “High risk of systemic infection progression (severe sepsis/septic shock). High risk for mortality after 30 day comparable with a SOFA score ≥ 8” [18, 19].

### Statistical analysis

Categorical data are reported as percentages and compared using Fisher’s exact test. Continuous data are reported as medians with inter-quartile ranges and compared using the Mann-Whitney U test. ROC curves were generated for presepsin values, inflammation-based prognostic scores, iPS, and Δpresepsin, and areas under the curve (AUCs), cut-off values, sensitivities, and specificities were calculated. For all values of presepsin, inflammation-based prognostic scores, iPS, and Δpresepsin, Kaplan-Meier curves were constructed for each mortality category, and the log-rank test was performed. Presepsin values on Day 1 and Day 2, inflammation-based prognostic scores, and iPS were examined further by multivariate logistic regression analysis for septic AKI, RRTi in sepsis patients, and prognosis in septic AKI patients. *P* < 0.05 was considered statistically significant. JMP software version 11.00 (SAS Institute Inc., NC, USA) was used for all statistical analyses.

## Results

Baseline characteristics of 83 adult patients diagnosed with sepsis according to the Sepsis-3 definition and admitted to the ICU are shown in Table 1. Median age was 74 years (range: 65.5–78.5). No significant differences were observed in age and sex in septic AKI patients and RRTi patients (Table 2).

**Table 1:** Baseline demographic characteristics

Variable	*n* = 83	
Age (years)	74.0	(65.5–78.5)
Sex (male) (%)	51.0	(61.4)
Cancer (%)	40.0	(48.2)
Coronary artery disease (%)	4.0	(4.8)
Diabetes mellitus (%)	10.0	(12.0)
Hypertension (%)	21.0	(25.3)
Albumin (g/dL)	2.3	(1.8–3.0)
CRP (mg/dL)	10.4	(3.7–17.5)
WBC (× 10^9^ l^−1^)	10.9	(5.4–15.4)
Neutrophil count (× 10^9^ l^−1^)	8.7	(3.56–13.29)
Lymphocyte count (× 10^9^ l^−1^)	0.5	(0.299–0.927)
Plt count (× 10^4^ mm^−3^)	17.8	(11.5–26.5)
Fibrinogen (mg/dL)	609.0	(378–711)
Survival (dead) (%)	26.0	(31.3)
AKI (%)	38.0	(45.8)
ARDS (%)	13.0	(15.7)
Shock (%)	48.0	(57.8)
DIC (%)	30.0	(36.1)
Presepsin on Day 1 (pg/mL)	1051.5	(569–1819.3)
Presepsin on Day 2 (ng/mL)	1016.5	(538–2156)
Presepsin on Day 3 (ng/mL)	802.0	(480.5–1825)
Presepsin on Day 5 (ng/mL)	1043.0	(480–1616)
ΔPresepsin Day 2 - Day 1 (pg/mL)	−21.50	(− 246.5–274.75)
ΔPresepsin Day 3 - Day 1 (pg/mL)	−38.50	(− 748.5–304)
ΔPresepsin Day 5 - Day 1 (pg/mL)	−59.50	(− 745.75–635.5)
GPS	1.0	(1–2)
NLR	12.6	(4.53–26.35)
PLR	321.9	(195.63–543.69)
PI	1.0	(1–2)
PNI	26.6	(21.26–33.72)
SOFA	8.0	(5–11)
qSOFA	2.0	(1–3)

*CRP* C-reactive protein, *WBC* white blood cell, *AKI* acute kidney injury, *ARDS* acute respiratory distress syndrome, *DIC* disseminated intravascular coagulation, *GPS* Glasgow Prognostic Score, *NLR* neutrophil to lymphocyte ratio, *PLR* platelet to lymphocyte ratio, *PI* Prognostic Index, *PNI* Prognostic Nutritional Index, *SOFA* Sequential Organ Failure Assessment, *qSOFA* quick Sequential Organ Failure Assessment

**Table 2:** Predictors of septic AKI and RRT initiation

	AKI (*n* = 38)	RRT initiation (*n* = 6)
	Univariate analysis	Univariate analysis
Variable	*P -*value	*P -*value
Age	0.402	0.108
Sex	0.930	0.670
Cancer	0.236	0.383
Coronary artery disease	0.621	none
Diabetes mellitus	0.005	0.620
Hypertension	0.010	0.368
Albumin	0.851	0.137
CRP	0.023	0.773
WBC	0.253	0.606
Neutrophil	0.315	0.564
Lymphocytes	0.631	0.127
Platelet count	0.081	0.127
Fibrinogen	0.427	0.088
Survival	0.052	0.333
ARDS	0.069	0.035
Shock	0.080	0.641
DIC	0.000	0.206
Presepsin on Day 1	0.001	0.149
Presepsin on Day 2	0.009	0.019
Presepsin on Day 3	0.143	0.905
Presepsin on Day 5	0.185	0.053
ΔPresepsin Day 2 - Day 1	0.810	0.032
ΔPresepsin Day 3 - Day 1	0.530	0.811
ΔPresepsin Day 5 - Day 1	0.408	0.053
GPS	0.232	0.832
NLR	0.969	0.837
PLR	0.032	0.458
PI	0.220	0.575
PNI	0.696	0.091
iPS-GPS	0.024	0.528
iPS-NLR	0.203	0.217
iPS-PLR	0.877	0.242
iPS-PI	0.025	0.782
iPS-PNI	0.172	0.718
SOFA	0.024	0.200
qSOFA	0.102	0.726

*AKI* acute kidney injury, *RRT* renal replacement therapy, *CRP* C-reactive protein, *WBC* white blood cell, *ARDS* acute respiratory distress syndrome, *DIC* disseminated intravascular coagulation, *GPS* Glasgow Prognostic Score, *NLR* neutrophil to lymphocyte ratio, *PLR* platelet to lymphocyte ratio, *PI* Prognostic Index, *PNI* Prognostic Nutritional Index, *iPS* inflammation-presepsin score, *SOFA* Sequential Organ Failure Assessment, *qSOFA* quick Sequential Organ Failure Assessment

There were 38 septic AKI patients defined as stage ≥1 according to the KDIGO classification, and 6 underwent RRTi after ICU admission. Of these, one patient withdrew from continuous renal replacement therapy, one patient’s end-stage kidney disease worsened, and four patients died during their ICU stay. ROC curve analyses revealed the following cut-off values (Table 3): AKI: 708.0 (pg/ml) for presepsin on Day 1 (AUC, 0.73; sensitivity, 81.6%; specificity, 58.5%) and 985.0 (pg/ml) for presepsin on Day 2 (AUC, 0.71; sensitivity, 65.5%; specificity, 64.3%); RRTi: 2014.0 (pg/ml) for presepsin on Day 1 (AUC, 0.71; sensitivity, 66.7%; specificity, 83.3%), 2867.0 (pg/ml) for presepsin on Day 2 (AUC, 0.90; sensitivity, 75.0%; specificity, 91.7%), 3014.0 (pg/ml) for presepsin on Day 5 (AUC, 0.96; sensitivity, 100.0%; specificity, 92.9%), 507.0 (pg/ml) for Δpresepsin (Day 2 – Day 1) (AUC, 0.84; sensitivity, 75.0%; specificity, 80.0%), 2385.0 (pg/ml) for Δpresepsin (Day 5 – Day 1) (AUC, 0.93; sensitivity, 100.0%; specificity, 93.3%), and 19.5 for PNI (AUC, 0.72; sensitivity, 66.7%; specificity, 93.5%).

**Table 3:** Receiver operating characteristic curve analysis

Variable	AUC	Cut-off	*P* value	Sensitivity (%)	Specificity (%)
AKI					
Presepsin on Day 1 (pg/mL)	0.73	708.00	*P* < 0.001	81.6	58.5
Presepsin on Day 2 (pg/mL)	0.71	985.00	0.002	65.5	64.3
RRT initiation					
Presepsin on Day 1 (pg/mL)	0.71	2014.00	0.155	66.7	83.3
Presepsin on Day 2 (pg/mL)	0.90	2867.00	P < 0.001	75.0	91.7
Presepsin on Day 5 (pg/mL)	0.96	3014.00	P < 0.001	100.0	92.9
ΔPresepsin Day 2 - Day 1 (pg/mL)	0.84	507.00	0.002	75.0	80.0
ΔPresepsin Day 5 - Day 1 (pg/mL)	0.93	2385.00	P < 0.001	1.0	93.3
PNI	0.72	19.51	0.145	66.7	93.5

*AUC* area under the curve, *AKI* acute kidney injury, *RRT* renal replacement therapy, *PNI* Prognostic Nutritional Index

Regarding prognosis in septic AKI patients, the results of ROC curve analyses, Log-rank test, and univariate analysis are shown in Tables 4, 5, and 6, respectively. ROC curve analyses revealed the following cutoff values: 28-day mortality: 1373.0 (pg/ml) for presepsin on Day 1 (AUC, 0.77; sensitivity, 81.8%; specificity, 76.9%), 1581.0 (pg/ml) for presepsin on Day 2 (AUC, 0.83; sensitivity, 85.7%; specificity, 68.2%), and 1.0 for iPS-PLR (AUC, 0.75; sensitivity, 90.9%; specificity, 46.2%); 60-day mortality: 1373.0 (pg/ml) for presepsin on Day 1 (AUC, 0.70; sensitivity, 75.0%; specificity, 76.0%), 1581.0 (pg/ml) for presepsin on Day 2 (AUC, 0.73; sensitivity, 75.0%; specificity, 66.7%), and 1.0 for iPS-PLR (AUC, 0.70; sensitivity, 83.3%; specificity, 44.0%); 90-day mortality: 1373.0 (pg/ml) for presepsin on Day 1 (AUC, 0.65; sensitivity, 64.3%; specificity, 73.9%) and 1581.0 (pg/ml) for presepsin on Day 2 (AUC, 0.65; sensitivity, 60.0%; specificity, 63.2%); 180-day mortality: 1336.0 (pg/ml) for presepsin on Day 1 (AUC, 0.65; sensitivity, 66.7%; specificity, 68.2%) and 1581.0 (pg/ml) for presepsin on Day 2 (AUC, 0.68; sensitivity, 63.6%; specificity, 66.7%) (Table 4). In the log-rank test, presepsin on Day 1 (*p* = 0.003), PNI (*p* = 0.001), and iPS-PLR (*p* = 0.036) were significant predictors of 28-day mortality, and presepsin on Day 1 (*p* = 0.007) and PNI (p = 0.003) were significant predictors of 60-day mortality (Table 5). In the univariate analysis, fibrinogen was a significant predictor of 28-day mortality (*p* = 0.0019), 60-day mortality (p = 0.0019), 90-day mortality (*p* = 0.0026), and 180-day mortality (*p* = 0.0043) (Table 6).

**Table 4:** Receiver operating characteristic curve analysis

Variable	AUC	Cut-off	P value	Sensitivity	Specificity
28-day mortality					
Presepsin on Day 1 (pg/mL)	0.77	1373.00	0.004	0.82	0.77
Presepsin on Day 2 (pg/mL)	0.83	1581.00	0.000	0.86	0.68
Presepsin on Day 3 (pg/mL)	0.91	1819.00	0.000	1.00	0.82
Presepsin on Day 5 (pg/mL)	1.00	3014.00	0.000	1.00	1.00
ΔPresepsin Day 2 - Day 1 (pg/mL)	0.76	507.00	0.059	0.71	0.86
ΔPresepsin Day 3 - Day 1 (pg/mL)	0.91	−10.00	0.000	1.00	0.76
ΔPresepsin Day 5 - Day 1 (pg/mL)	0.79	2385.00	0.176	0.75	1.00
iPS-PLR	0.75	1.00	0.002	0.91	0.46
60-day mortality					
Presepsin on Day 1 (pg/mL)	0.70	1373.00	0.063	0.75	0.76
Presepsin on Day 2 (pg/mL)	0.73	1581.00	0.052	0.75	0.67
Presepsin on Day 3 (pg/mL)	0.75	1819.00	0.134	0.80	0.81
Presepsin on Day 5 (pg/mL)	0.80	3014.00	0.134	0.80	1.00
ΔPresepsin Day 2 - Day 1 (pg/mL)	0.74	507.00	0.045	0.63	0.86
ΔPresepsin Day 3 - Day 1 (pg/mL)	0.85	−10.00	0.000	0.80	0.75
ΔPresepsin Day 5 - Day 1 (pg/mL)	0.72	2385.00	0.238	0.60	1.00
iPS-PLR	0.70	1.00	0.024	0.83	0.44
90-day mortality					
Presepsin on Day 1 (pg/mL)	0.65	1373.00	0.142	0.64	0.74
Presepsin on Day 2 (pg/mL)	0.65	1581.00	0.190	0.60	0.63
Presepsin on Day 3 (pg/mL)	0.74	1545.00	0.085	0.83	0.73
Presepsin on Day 5 (pg/mL)	0.79	1399.00	0.082	0.83	0.73
ΔPresepsin Day 2 - Day 1 (pg/mL)	0.69	507.00	0.092	0.50	0.84
ΔPresepsin Day 3 - Day 1 (pg/mL)	0.87	−10.00	0.000	0.83	0.80
ΔPresepsin Day 5 - Day 1 (pg/mL)	0.74	244.00	0.108	0.67	0.82
180-day mortality					
Presepsin on Day 1 (pg/mL)	0.65	1336.00	0.121	0.67	0.68
Presepsin on Day 2 (pg/mL)	0.68	1581.00	0.093	0.64	0.67
Presepsin on Day 3 (pg/mL)	0.80	1545.00	0.019	0.86	0.79
Presepsin on Day 5 (pg/mL)	0.77	1313.00	0.063	0.86	0.70
ΔPresepsin Day 2 - Day 1 (pg/mL)	0.73	507.00	0.022	0.55	0.89
ΔPresepsin Day 3 - Day 1 (pg/mL)	0.91	−10.00	0.000	0.86	0.86
ΔPresepsin Day 5 - Day 1 (pg/mL)	0.76	−23.00	0.051	0.71	0.80

*AUC* area under the curve, *iPS* inflammation-presepsin score, *PLR* platelet to lymphocyte ratio

**Table 5:** Log-rank test

Variable	P value
28-day mortality	
Presepsin on Day 1 (pg/mL)	0.00
Presepsin on Day 2 (pg/mL)	0.086
Presepsin on Day 3 (pg/mL)	0.126
Presepsin on Day 5 (pg/mL)	0.002
ΔPresepsin Day 2 - Day 1 (pg/mL)	0.014
ΔPresepsin Day 3 - Day 1 (pg/mL)	0.093
ΔPresepsin Day 5 - Day 1 (pg/mL)	0.008
PNI	0.001
iPS - PLR	0.036
60-day mortality	
Presepsin on Day 1 (pg/mL)	0.007
Presepsin on Day 2 (pg/mL)	0.170
Presepsin on Day 3 (pg/mL)	0.367
Presepsin on Day 5 (pg/mL)	0.003
ΔPresepsin Day 2 - Day 1 (pg/mL)	0.026
ΔPresepsin Day 3 - Day 1 (pg/mL)	0.223
ΔPresepsin Day 5 - Day 1 (pg/mL)	0.009
PNI	0.003
90-day mortality	
Presepsin on Day 1 (pg/mL)	0.056
Presepsin on Day 2 (pg/mL)	0.788
Presepsin on Day 3 (pg/mL)	0.212
Presepsin on Day 5 (pg/mL)	0.226
ΔPresepsin Day 2 - Day 1 (pg/mL)	0.090
ΔPresepsin Day 3 - Day 1 (pg/mL)	0.091
ΔPresepsin Day 5 - Day 1 (pg/mL)	0.072
180-day mortality	
Presepsin on Day 1 (pg/mL)	0.079
Presepsin on Day 2 (pg/mL)	0.798
Presepsin on Day 3 (pg/mL)	0.128
Presepsin on Day 5 (pg/mL)	0.265
ΔPresepsin Day 2 - Day 1 (pg/mL)	0.038
ΔPresepsin Day 3 - Day 1 (pg/mL)	0.029
ΔPresepsin Day 5 - Day 1 (pg/mL)	0.057

*PNI* Prognostic Nutritional Index, *iPS* inflammation-presepsin score, *PLR* platelet to lymphocyte ratio

**Table 6:** Predictors of mortality in sepsis patients (univariate analysis)

	28-day mortality	60-day mortality	90-day mortality	180-day mortality
Variable	*P-*value	*P-*value	*P-*value	*P-*value
Age	0.4744	0.6260	0.3081	0.4572
Sex	0.7277	1.0000	1.0000	0.7341
Cancer	0.1691	0.3193	0.4979	0.7384
Coronary artery disease	1.0000	1.0000	1.0000	1.0000
Diabetes mellitus	0.2293	0.2204	0.4339	0.2616
Hypertension	0.4657	0.2863	0.7379	0.5144
Albumin	0.1570	0.4257	0.2720	0.5150
CRP	0.1579	0.0535	0.1068	0.1417
WBC	0.4156	0.4654	0.9625	0.8406
Neutrophil	0.4252	0.3468	0.8510	0.9753
Lymphocytes	0.3352	0.7212	0.7541	0.9261
Platelet count	0.1183	0.2699	0.3721	0.3147
Fibrinogen	0.0019	0.0019	0.0026	0.0043
ARDS	0.1249	0.0486	0.1322	0.2578
Shock	0.7217	0.7110	0.7351	0.7235
DIC	0.2847	0.4912	1.0000	1.0000
Presepsin on Day 1	0.0115	0.0556	0.1368	0.1257
Presepsin on Day 2	0.0093	0.0570	0.1909	0.1105
Presepsin on Day 3	0.0122	0.0986	0.0868	0.0305
Presepsin on Day 5	0.0032	0.0578	0.0562	0.0637
ΔPresepsin Day 2 - Day 1	0.0415	0.0454	0.1033	0.0366
ΔPresepsin Day 3 - Day 1	0.0122	0.0208	0.0102	0.0028
ΔPresepsin Day 5 - Day 1	0.0894	0.1706	0.1078	0.0790
GPS	0.5179	0.2002	0.5073	0.7101
NLR	0.3030	0.1194	0.3638	0.4036
PLR	0.7649	0.7952	0.8756	0.5990
PI	0.1751	0.1579	0.6093	0.9329
PNI	0.0911	0.4362	0.3014	0.5777
iPS-GPS	0.1270	0.3206	0.3377	0.3436
iPS-NLR	0.1220	0.2898	0.2913	0.4036
iPS-PLR	0.0100	0.0365	0.0840	0.1932
iPS-PI	0.2891	0.5490	0.3714	0.2989
iPS-PNI	0.7178	0.1765	0.4710	0.5194
SOFA	0.8806	0.6250	0.3067	0.4653
qSOFA	0.8457	0.5805	0.2173	0.4010

*CRP* C-reactive protein, *WBC* white blood cell, *AKI* acute kidney injury, *ARDS* acute respiratory distress syndrome, *DIC* disseminated intravascular coagulation, *GPS* Glasgow Prognostic Score, *NLR* neutrophil to lymphocyte ratio, *PLR* platelet to lymphocyte ratio, *PI* Prognostic Index, *PNI* Prognostic Nutritional Index, *iPS* inflammation-presepsin score, *SOFA* Sequential Organ Failure Assessment, *qSOFA* quick Sequential Organ Failure Assessment

We also performed multivariate logistic regression analyses to identify independent predictors of septic AKI, RRTi in septic patients, and prognosis in septic AKI patients (Table 7). Multivariate logistic regression analyses revealed that presepsin on Day 2 is a predictor of prognosis in septic AKI patients.

**Table 7:** Multivariate analysis

Variable (examined explanatory variable)	Odds ratio	95% CI		*P* value
28-day mortality (Presepsin on Day 1, Presepsin on Day 2)				
Presepsin on Day 1	0.9989	0.9974	1.0003	0.120
Presepsin on Day 2	1.0018	1.0002	1.0034	0.027
28-day mortality (Presepsin on Day 1, iPS-PLR)				
Presepsin on Day 1	1.0004	0.9998	1.0010	0.182
iPS-PLR	3.1006	0.7548	12.7370	0.116
28-day mortality (Presepsin on Day 1, Cre on Day 1)				
Presepsin on Day 1	1.0006	1.0001	1.0012	0.028
Cre on Day 1	0.6670	0.3102	1.4342	0.300
28-day mortality (Presepsin on Day 2, Cre on Day 2)				
Presepsin on Day 2	1.0008	1.0000	1.0015	0.053
Cre on Day 2	0.5674	0.1051	3.0628	0.510
60-day mortality (Presepsin on Day 1, Presepsin on Day 2)				
Presepsin on Day 1	0.9989	0.9975	1.0003	0.132
Presepsin on Day 2	1.0015	1.0001	1.0028	0.039
60-day mortality (Presepsin on Day 1, iPS-PLR)				
Presepsin on Day 1	1.0003	0.9998	1.0009	0.217
iPS-PLR	2.1516	0.5980	7.7414	0.241
60-day mortality (Presepsin on Day 1, Cre on Day 1)				
Presepsin on Day 1	1.0005	1.0000	1.0010	0.054
Cre on Day 1	0.5945	0.2704	1.3070	0.196
60-day mortality (Presepsin on Day 2, Cre on Day 2)				
Presepsin on Day 2	1.0006	1.0000	1.0012	0.058
Cre on Day 2	0.4995	0.1077	2.3167	0.375
90-day mortality (Presepsin on Day 1, Presepsin on Day 2)				
Presepsin on Day 1	0.9991	0.9978	1.0004	0.172
Presepsin on Day 2	1.0011	0.9998	1.0023	0.086
90-day mortality (Presepsin on Day 1, Cre on Day 1)				
Presepsin on Day 1	1.0004	0.9999	1.0009	0.104
Cre on Day 1	0.6748	0.3491	1.3044	0.242
90-day mortality (Presepsin on Day 2, Cre on Day 2)				
Presepsin on Day 2	1.0004	0.9999	1.0009	0.100
Cre on Day 2	0.6546	0.2224	1.9265	0.442
180-day mortality (Presepsin on Day 1, Presepsin on Day 2)				
Presepsin on Day 1	0.9987	0.9972	1.0002	0.078
Presepsin on Day 2	1.0015	1.0001	1.0029	0.037
180-day mortality (Presepsin on Day 1, Cre on Day 1)				
Presepsin on Day 1	1.0004	0.9999	1.0009	0.124
Cre on Day 1	0.6812	0.3612	1.2850	0.236
180-day mortality (Presepsin on Day 2, Cre on Day 2)				
Presepsin on Day 2	1.0004	0.9999	1.0010	0.100
Cre on Day 2	0.6311	0.2145	1.8566	0.403

*CI* confidence interval, *iPS* inflammation-Presepsin Score, *PLR* platelet to lymphocyte ratio, *Cre* creatinine

## Discussion

Sepsis involves lethal organ dysfunction due to the activation of both pro- and anti-inflammatory responses [20], and is modified by non-immunologic pathways, including cardiovascular, neuronal, autonomic, hormonal, bioenergetic, metabolic, and coagulation pathways [21–23]. Severe sepsis is associated with a mortality rate of > 50% [24], and the most common cause of AKI among critically ill patients is sepsis [3]. PCT has the highest specificity among diagnostic markers for sepsis, but can yield false positive results since its levels increase in various non-sepsis contexts (e.g., severe trauma, invasive surgical procedure, critical burn injuries) [8–10]. Presepsin, another diagnostic marker for sepsis, is secreted from granulocytes in response to infectious stimuli in an animal sepsis model [25]. According to Liu et al., presepsin was the best predictor of early stage sepsis in emergency department patients [26]. Presepsin values have also been reported to be associated with organ dysfunction, coagulation disorders, and ICU mortality [27].

In the present study, presepsin cut-off values for predicting septic AKI, RRTi in sepsis patients, and prognosis in septic AKI patients were higher than those previously reported as predicting severe sepsis and septic shock [18, 19] (Tables 3 and 4). Moreover, presepsin values for predicting RRTi had a higher cut-off value and specificity relative to those for predicting septic AKI. A significant negative correlation was previously reported between presepsin levels and estimated glomerular filtration rate in both non-sepsis and sepsis patients [28]. Increases in presepsin levels in hemodialysis (HD) patients may not be related to renal dysfunction, but rather the activation of neutrophils and/or monocytes, since HD activates monocytes and/or neutrophils, which in turn leads to presepsin release from monocytes [29]. Presepsin levels in HD patients without infection were reported to be 783–2360 pg/ml [30]. Nakamura et al. retrospectively examined presepsin values in ICU patients with or without sepsis, and reported that presepsin values were markedly high in patients with renal failure and end-stage kidney disease. Presepsin values in those with sepsis ranged from 2632 to 20,000 pg/ml, while patients without sepsis had presepsin values of 2134 to 19,633 pg/ml [28]. In the present study, presepsin cut-off values for predicting RRTi were similar to these previously reported levels. Our results suggest the need to adopt a higher presepsin cut-off value for predicting septic AKI, RRTi in sepsis patients, and prognosis in septic AKI patients.

Cut-off values for predicting septic AKI, RRTi in sepsis patients, and prognosis in septic AKI patients on Day 2 or later after ICU entry were higher than those on Day 1 (Tables 3 and 4). Multivariate logistic regression analyses identified presepsin on Day 2 to be a predictor of prognosis in septic AKI patients (Table 7). Presepsin levels measured at the time of ICU admission may not be at an optimal level for predicting septic AKI, RRTi in sepsis patients, and prognosis in septic AKI patients. Indeed, several studies have reported that presepsin levels increase as the severity of sepsis increases [13, 26, 31]. For instance, Masson et al. reported that an increase in presepsin levels from Day 1 to Day 2 after ICU admission can predict higher ICU and 90-day mortality [27]. Our findings suggest the importance of not only measuring presepsin levels at the time of ICU admission, but also monitoring its temporal changes after ICU admission in order to better predict the onset of septic AKI, RRTi in sepsis patients, and prognosis in septic AKI patients. Our findings may also provide insight on the optimal timing for RRTi in septic AKI patients.

Presepsin values increase with declining renal function [30], suggesting that the diagnostic accuracy of presepsin may be influenced by renal function. Multivariate analyses revealed that serum creatinine immediately after ICU admission (baseline) and on Day 2 were not predictors of 28-day, 60-day, 90-day, or 180-day mortality (Table 7). In contrast, presepsin immediately after ICU admission (baseline) and on Day 2 were significant predictors of 28-day mortality, even after adjusting for serum creatinine immediately after ICU admission (baseline) and on Day 2 (Table 7). These findings suggest that renal function had a minimal, if any, impact as a confounder. However, presepsin cannot be used as a single definitive index to diagnose the prognosis associated with sepsis. Thus, in addition to using presepsin, critical care physicians must comprehensively evaluate the clinical findings of each patient and make a diagnosis based on all information available.

The AUC, sensitivity, and specificity of PNI for predicting RRTi were 0.72, 66.7, and 93.5%, respectively, with a higher specificity than that for presepsin alone on Days 1, 2, and 5. The corresponding median PNI values (inter-quartile range) were 18.4 (14.6–30.0) in RRTi patients and 28.9 (22.7–34.1) in non-RRTi patients. These findings suggest that a lower PNI can predict RRTi in sepsis patients and may serve as an easy “rule in” test at the time of ICU admission. PNI can be obtained at low cost and rapidly in clinical settings where presepsin values cannot be easily measured, and provides information necessary for interventions in sepsis patients within the first few hours of ICU admission. Our findings also suggest that hypoalbuminemia and lymphocytopenia (albumin and lymphocyte counts are used to calculate PNI) are important variables for predicting RRTi in sepsis patients. Serum albumin levels are significantly correlated with presepsin levels [30]. Zahorec et al. found a correlation between severity of the clinical course and extent of lymphocytopenia in oncological ICU patients following major surgery, sepsis, and septic shock [32].

The univariate analysis revealed fibrinogen to be a significant predictor of mortality in septic AKI patients (Table 6). In the clinical setting, elevated plasma fibrinogen levels are used to predict progression or poor outcome in patients with several types of malignancies, including esophageal cancer [33], gastric cancer [34], pancreatic cancer [35], colon cancer [36], lung cancer [37], and gynecological cancer [38–40]. Elevated plasma fibrinogen might serve as a useful inflammation-based prognostic biomarker in septic AKI patients.

This study has several limitations. First, the present study was conducted at a single center with a small sample size. Second, we used a single biomarker, and no comparisons were made with other biomarkers.

## Conclusions

Presepsin and PNI were found to be predictors of septic AKI, RRTi in sepsis patients, and prognosis in septic AKI patients. Cut-off values and specificities for predicting septic AKI and RRTi on Day 2 or later were higher than those on Day 1. Further studies aimed at understanding the exact role of presepsin values and PNI in predicting septic AKI, RRTi in sepsis patients, and prognosis in septic AKI patients are warranted.

## Data Availability

The datasets used and/or analyzed during the current study are available from the corresponding author on reasonable request.

## Abbreviations

GPS: Glasgow Prognostic Score
CRP: C-reactive protein
NLR: Neutrophil to lymphocyte ratio
PLR: Platelet to lymphocyte ratio
PNI: Prognostic Nutritional Index
PI: Prognostic Index
SOFA: Sequential Organ Failure Assessment
qSOFA: Quick Sequential Organ Failure Assessment
ROC: Receiver operating characteristic
AUC: Area under the curve
RRTi: Renal replacement therapy initiation
AKI: Acute kidney injury
HD: Hemodialysis
